# Ending the HIV epidemic PrEP equity recommendations from a rapid ethnographic assessment of multilevel PrEP use determinants among young Black gay and bisexual men in Atlanta, GA

**DOI:** 10.1371/journal.pone.0283764

**Published:** 2023-03-30

**Authors:** Miranda Hill, Justin Smith, Dena Elimam, Genetha Mustafaa, Pascale Wortley, Brittany Taylor, Orlando Harris

**Affiliations:** 1 School of Medicine, University of California, San Francisco, San Francisco, California, United States of America; 2 Positive Impact Health Centers, Atlanta, Georgia, United States of America; 3 Georgia HIV Behavioral Surveillance, Georgia Department of Public Health, Atlanta, Georgia, United States of America; 4 Georgia State University, Atlanta, Georgia, United States of America; 5 School of Nursing, University of California, San Francisco, San Francisco, California, United States of America; College of Medicine of the University of Lagos, NIGERIA

## Abstract

**Background:**

Efforts to mitigate HIV transmission among gay and bisexual men have not been sufficient to level persistent racial inequities which now extend to the use of daily oral pre-exposure prophylaxis (PrEP) for HIV prevention. Community-involved ethnographic research is crucial to galvanizing collaboration between patients, researchers, and policymakers to identify the social determinants of emerging PrEP inequities. In partnership with community key informants, we conducted a Rapid Ethnographic Assessment (REA) of multilevel PrEP use determinants among young Black gay and bisexual (YBGBM) men in the metropolitan Atlanta area to inform the development and coordination of local HIV programs.

**Methods:**

In the assessment, we drew upon the perspectives of local clinicians, community-based organization leaders, health educators, and PrEP clients to identify barriers and facilitators to PrEP use among YBGBM through interviews (N = 23). Data were collected from September 2020 –to January 2021 and were analyzed through a staged deductive-inductive thematic analysis. The themes were later summarized and presented to community stakeholder participants to facilitate member-checking.

**Results:**

Our analyses revealed structural, cultural, relationship, and developmental factors which shaped PrEP use. The most prominent being “ease of access to PrEP” (structural level), “provider support” (interpersonal), and “life-stage traits” (individual). Our results also contribute novel information concerning the axes of intersectional stigma (spatial, racial, sexual identity, and HIV) among YBGBM in Atlanta and its divergent effects on PrEP use.

**Conclusion:**

Increased PrEP use among YBGBM, particularly among those living in the south, is essential to ending the domestic HIV epidemic. Altogether, our results emphasize the need for PrEP program modifications, which increase flexibility in methods and modes of access and are culturally adapted to the needs of YBGBM. There is also a need for resources that holistically focus on mental health, trauma, and racism as critical components of support.

## Introduction

Gay and bisexual men (GBM) are disproportionately affected by the HIV/AIDS epidemic in the United States. According to the Centers for Disease Control and Prevention (CDC), it is estimated that male-to-male sexual contact accounted for 69% of new HIV infections among adult and adolescent males in the United States in 2019 [1]. In 2019, young GBM between the ages of 13–24 years accounted for 21% of new HIV infections among youth in that age group [2]. African American or Black GBM have sustained a higher burden of the HIV epidemic for decades. In 2019, approximately 42% of HIV infections were among Black GBM, and nearly half (51%) of HIV infections among GBM, ages 13–24 years, were among Black GBM [3].

There are also fundamental regional differences in the distribution of HIV across the US. In 2018, the US south had more HIV diagnoses (19, 396) than any other region [4], with the majority among Black GBM. Racial inequities are pronounced in the south, where 56% of the US Black population resides [5]. Atlanta, Georgia is the most populous metropolitan area of residence in the south for Black/African Americans and a site with a significant burden of HIV among GBM [5, 6]. It is estimated that nearly 72% of HIV transmission cases among males in Atlanta are attributable to male-to-male sexual contact and the rate of Black males living with HIV is nearly 6 times that of white males [6].

Increasing the uptake and use of Pre-exposure prophylaxis (PrEP) among high HIV-burden groups in high-HIV-burden geographic areas is a key prevention strategy within the Ending the HIV Epidemic plan (EHE). The EHE plan is a cross-agency federal operational initiative in the U.S. that began in 2019 with the end goal of “reducing new HIV infections in the United States by 75% by 2025 and 90% by 2030 [7].” PrEP is an antiviral medicine that, when taken as prescribed, can markedly reduce HIV acquisition among HIV-negative people prior to exposure by about 99% [8, 9]. Though PrEP has been recently approved to be administered to people at risk for HIV through sex by health providers in the form of an injection, the original and most widely available PrEP modality is a daily oral pill regimen that requires high levels of adherence and continuous health care engagement for maximum clinical effectiveness [10]. Moreover, there is some data from a study with a racially diverse sample of GBM that suggests that despite the convenience and lower adherence barriers with injectable PrEP, daily oral PrEP will likely remain a preferred HIV prevention modality among people who dislike needles and are concerned about side effects, and treatment efficacy [11]. Eliminating barriers to daily oral PrEP use among Black gay and bisexual men, particularly, those living in geographic areas with dense HIV inequities, (including the Atlanta metropolitan area) continues to be prioritized within the Ending the HIV Epidemic (EHE) plan) [7] A complex array of factors have been implicated as barriers to daily oral PrEP use among various subgroups of GBM [12–14]. Research and surveillance data indicate that young Black gay and bisexual men in the southern U.S. may encounter significant barriers to daily oral PrEP access and use, above and beyond other groups [12, 13]. Prescribing data suggests that the majority of PrEP prescriptions are given to cisgender women, followed by white gay and bisexual men [15, 16]. Additional data indicate lower PrEP uptake and usage among younger people, Black/African Americans, and people living in the south [13, 14, 17]. For example, a recent federal surveillance survey found that Black GBM were least likely to be aware of PrEP, discuss PrEP with a provider, or have used PrEP in the past year [17] Moreover, the survey results indicate that inequities persist between Black and White GBM participants beyond awareness, whereby 68% of White GBM who were aware of PrEP were also using it, while only 55% of Black GBM who were aware of PrEP were using PrEP [17]. Despite predictions of broadening HIV inequities between Black gay men and other groups due to disparate PrEP use trends and an imminent need to inform the development of culturally-relevant EHE strategies, significant knowledge gaps remain concerning context- and population-specific PrEP use barriers and facilitators among YBGBM [17].

The present study seeks to address the above knowledge gaps by answering the following question: *what are multilevel determinants of PrEP use among YBGBM in Atlanta*? We used a rapid ethnographic assessment (REA) to foster a collaborative research-community-partnered approach to answering the above research question while achieving our primary objective of generating actionable insights for stakeholders developing local EHE responses. REAS are short-term qualitative research projects expedient to investigating how and/or why a problem is occurring and how to best respond. They are frequently employed when, 1) more information is needed about a problem, 2) a particular issue is evolving, 3) there is a need to reach medically underserved groups, 4) program or policy adjustments are needed, 5) there is a need to involve the community, and finally, 6) limited time and resources are available to rapidly inform public health decision-making [18]. REAs are rooted in anthropological traditions that centralize knowledge production within communities. Thus, REAs are simultaneously driven and informed by local populations. REAs are also unique from other forms of qualitative research, given the explicit focus on developing recommendations to promptly guide responses that address community needs [19, 20]. The REA herein was prompted amid the planning and distribution of federal EHE resources to the metropolitan-Atlanta region.

Synthesized theories on social structure, inequality, and population health framed the investigation. Ecological models are widely used to organize multilevel examinations of social and structural behavioral health determinants [21]. We used Bronfenbrenner’s socioecological model to organize the examination of PrEP use barriers and facilitators on the individual (e.g., individual behaviors), interpersonal (e.g., relationship factors), community (e.g., proximal networks, environmental factors), and structural/societal levels (e.g., organizational policies and factors) [22]. Intersectionality theory, which focuses attention on the role of structural power and policies in creating variable social disadvantages [23] is also applied here as a lens and analytical frame for examining the social construction of PrEP inequities among YBGBM in Atlanta, GA due to structural inequalities among marginalized groups at the intersection of race, gender, and place. These theories, combined, serve as an integrated framework for exploring our research question which is focused on exploring the dynamic interplay between the multilevel determinants that shape daily oral PrEP access and utilization among YBGBM through the perspectives of PrEP clinicians, administrators, and clients.

## Materials and methods

### Recruitment

Participants were purposively sampled for study inclusion. Purposive sampling strategies are commonly used in REAs, and other forms of qualitative research given the emphasis on recruiting key informants who are most knowledgeable about a particular population or subject. Recruitment occurred in two successive, and eventually, overlapping phases, beginning with providers in the metropolitan Atlanta region. In this study, “providers” were PrEP coordinators, clinicians, administrators, and/or community liaisons involved in the development and delivery of PrEP and/or community-based support services to YBGBM in Atlanta. Provider recruitment (from September 2020 to January 2021) began with sending email invitations or placing phone calls to individuals and organizations who formerly participated in HIV prevention research with gay and bisexual men. Additional providers were recruited through snowball sampling, whereby those who participated in interviews referred others who also worked closely with the community. PrEP user participants were recruited from December 2020 to January 2021 through local providers and social organizations. Specifically, recruitment emails with flyers describing the study, eligibility criteria, and incentives were delivered to providers to share within their networks and affiliated organizations. PrEP user participants were screened for eligibility over the phone. Eligible participants 1) identified as Black/African American, 2) were 18–29 years of age, 3) identified as gay or bisexual, 4) had previously had oral or anal sex with another man, 5) had been prescribed PrEP, and 6) had accessed PrEP services in Metro-Atlanta area within the past two years.

### Data collection

Prior to data collection, interviewers read the consent form aloud to participants and documented verbal consent with a designated check box located on the researchers’ copy of consent form. Those who verbally consented “yes” to participate in the study were enrolled in interviews. All provider interviews (N = 23) were conducted by the study ethnographer (M.H.), who is a Black woman and social and behavioral scientist, trained and experienced in conducting community-based HIV services evaluation research. During the interviews, the ethnographer asked questions about providers’ role in PrEP/HIV service provision, local observations, perceptions of PrEP use barriers and facilitators among YBGBM, and information gaps to explore in PrEP client interviews. The provider interviews, which were not audio-recorded, lasted for 30 minutes on average. Providers were not incentivized for their time. Extensive field notes were taken to record interview content, with points of emphasis and key statements being recorded verbatim.

PrEP use determinants among YBGBM were explored through in-depth individual interviews with PrEP clients. The semi-structured interview guide used to facilitate the interviews was initially framed by intersectionality theory with the socioecological model [22, 24]. The specific questions were further tailored to address the informational needs and knowledge gaps presented by provider participants. PrEP user interviews were conducted by a trained and experienced White Queer sociologist (B.T.) and the study ethnographer (M.H.). The audio-recorded interviews lasted for about an hour. Participants were compensated with $50 in gift cards. Field notes were recorded after the interviews to summarize and highlight observations. An external transcription agency transcribed the interviews. The provider and PrEP user interviews notes and transcripts were imported into the Dedoose 8.3 software package for storage, management, and analyses. All study procedures were approved by the Institutional Review Board at the Georgia Department of Public Health.

### Data analyses

Data from provider and client interviews were analyzed iteratively through a deductive-inductive thematic analysis [25]. The deductive approach to analysis began with the development of a skeleton coding template based on theory and research questions. The interviewers (M.H. & B.T.) were most familiar with the data and thus debriefed the team during weekly meetings beginning with data from fieldnotes and direct quotations highlighting investigation areas of emphasis concerning provider observations and information gaps. The results from an initial rapid thematic analysis of provider data were then integrated into the client interview guide for the purpose of developing and tailoring research questions. Preliminary data from provider interviews were also applied to the development of a coding template that would be used in a more rigorous hybrid deductive-inductive analysis of provider and client interviews that was initiated after client interviews started. Concurrent with the remainder of data collection from providers and clients, M.H. and B.T. discussed observations and contemplated commonalities and discrepancies with project members during weekly team meetings. Raw data, observations, and meeting notes drove the development of a code template that was applied to the data. Data were reviewed and explored for patterns and emergent themes as dictated by the repetition, forcefulness, and co-occurrence of codes as they related to the PrEP use determinants [26]. Deliberation amongst the team fostered theme development and the triangulation of data sources, thereby strengthening the validity of findings. After the analyses concluded, the themes were organized into matrix tables (organized by the socioecological framework and participant type) and presented in summary form to community stakeholder participants. This efficient member-checking process allowed us to draw upon perspectives to challenge, verify, and translate findings into actionable recommendations.

## Results

The participants (N = 23) were diverse in their roles and perspective on multilevel PrEP use determinants among YBGBM. Descriptive demographic data were limited due to protocol and time restrictions. Table 1 describes the sample according to their role and organizational affiliation.

**Table 1:** Description of participant roles.

Participant Roles	N
Community-based organization leader	2
Community health outreach worker	2
Clinical PrEP Coordinator	3
Public Health Educator	1
HIV Program Administrator	2
Nurse	1
Clinical PrEP promotion team	5
PrEP Clients	7

Most interviews were conducted remotely through phone or videoconference (N = 21) due to a scarcity of protective gear for in-person operations (due to a pandemic of the SARS-CoV-2 virus, which causes COVID-19). In-person observations were additionally restricted by organizational protocols which limited on-site operations for providers. In all, we conducted 14 individual and 3 group-based interviews during the assessment. The 3 group-based interviews each consisted of 2–5 providers who volunteered to participate in the interviews jointly. All interviews with clients were individual, private, and remote.

**Fig 1:**
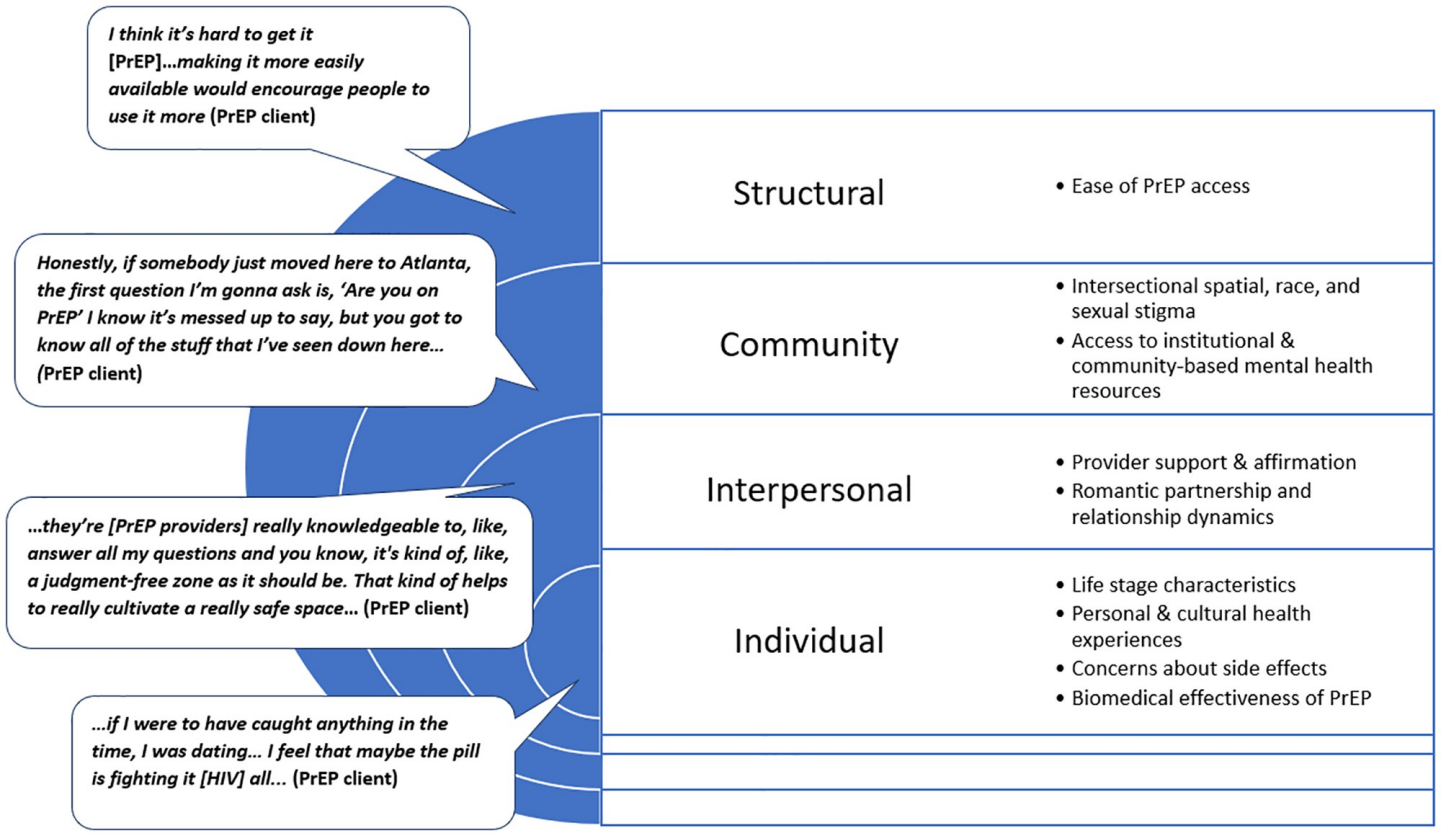
Socioecological model of rapid ethnographic assessment themes with exemplary quotes.

Our analyses revealed PrEP access and utilization determinants among YBGBM in Atlanta across multiple structural levels. The following sections organize findings beginning with upstream determinants, while also highlighting the interaction between factors operating on multiple levels. The primary themes from the analyses are summarized and presented in Fig 1. We assigned pseudonyms to protect participant identities.

### Structural level

#### Ease of PrEP access

Ease of PrEP access (broadly defined as the financial, physical, time, and social costs associated with PrEP prescriptions and care) was a prevalent structural determinant presented in discussions surrounding describing where, when, and how YBGBM could access PrEP in Atlanta. Participants’ consideration of which factors made accessing PrEP easy or challenging varied according to their resources and preferences. While some felt that getting their PrEP prescriptions from the pharmacy and engaging in clinical PrEP care was “easy,” others considered going to the clinic to be inconvenient and prohibitive. For example, when asked about what he liked about PrEP, one participant described the simplicity of clinical access, stating:

*It’s literally in and out…they start, you know, taking a little basic information, checking your vitals and stuff…there is really no excuse for why… not to take it*.

(Corey)

In contrast, another participant emphasized the ease of receiving PrEP through a mail program. Specifically, when asked *is there anything else*, *at any point of accessing PrEP that works for you*? he replied, “*The way I have gotten it*.” He went on to explain why he “loved” the PrEP mail delivery program that he found on social media, while also highlighting how such programs could work for people with clinical access barriers:

*“It was mailed to me*, *like*, *the process was really smooth*. *I think that works for me the most… So*, *there was one time I did do a test and it came back positive for chlamydia and it was something I didn’t even know…So*, *yeah*, *they treated me for that*, *and they also gave me PrEP stuff…I love the process*.

(Brandon)

Mail delivery programs may also ease access to PrEP among those who are deterred from clinical PrEP care due to stigma. Clients and providers emphasized the prohibitive effects of HIV stigma on PrEP-seeking among YBGM at HIV treatment facilities. One participant described the impacts of HIV stigma on his friends:

*They don’t want their cars to be seen at a clinic and stuff like that [because] stigma and*, *you know*, *maybe some of those… those personal*, *you know*, *psychological barriers to taking that next step*.

(Corey)

While discussing barriers to care, a provider involved in the PrEP program at an HIV clinic stated:

*People are afraid of being seen going into the clinic by others who might tell other people that they are living with HIV*

(Provider A).

Clinical hours and perceived medical expenses were other factors that influenced the ease of PrEP access. In a discussion about cost-related barriers to PrEP use among YBGM, one provider stated, *we try to remove barriers such as the costs of visits*, *labs*, *etc*. (Provider B). A provider at a different organization agreed, stating:

*99% of the time*, *the issue isn’t with insurance or cost for the clients that I see*.

(Provider A)

Yet, clients often mentioned being surprised by the affordability of PrEP after initiating care, given preconceived notions about high costs. In response to being asked about barriers to PrEP use among YBGBM in Atlanta, one stressed the need for increased public education about free or low-cost PrEP in Atlanta:

*The medication is really expensive…you know, there’s a lot of free programs now for it, but a lot of people don’t know about that…I think it should be a lot more publicized that you can get it completely free*.

(Devon)

When asked about his experiences navigating local health care systems, another participant described how important financial assistance programs and integrated patient care were to him. When asked about how he accessed PrEP, he replied:

*That right there was*, *um*, *a big thing for me*. *Accessibility…they have a discount card for a lot of people who don’t have insurance …they can help with financial assistance… It just made a big difference just to have that unity working in healthcare*, *knowing that us*, *black folks*, *can be*, *you know*, *put*, *put to the end of the*, *um*… *the back of the bus or we get the short end of the stick a lot of the times*.

(Jay).

### Community

#### Intersectional stigma

Analyses of community-level determinants revealed that HIV stigma (as manifested on the structural level) most often intersected with racism and homophobia (e.g., Black gay man) and spatial stigma (defined by negative representations of specific locales and their inhabitants [27]). The impacts of the intersectional stigma associated with being a “Black gay man in Atlanta,” on PrEP use varied, whereby the impacts deterred PrEP access among some (as described in the previous section), while alternatively, motivating PrEP adherence among others.

For example, when asked about PrEP use after moving to Atlanta from a major city in the Northeast, one such participant replied:

*I’m better with it now…I know it sounds bad*, *but specifically living*, *like*, *in Atlanta*, *and the high HIV rate*, *I’m like*, *let me just be as safe as possible… The knowing of rates before I came… I don’t know if it’s a global stigma against Atlanta about the whole HIV*, *everything going on here*. *But before I came here…you know*, *people around me*, *peers*, *would be like*, *‘If you’re going to Atlanta*, *make sure you just stay on it [PrEP]*,*’ etcetera*, *etcetera…I said*, *‘Let me just make sure that I stay as healthy as possible…*

(Lamar)

Similarly, another described how identity intersected with the spatial stigmatization of Atlanta to promote PrEP use, stating:

…*I am a black*, *gay male in Atlanta*. *Of course…I want to have sex just like everybody else*. *But*, *at the same time*, *I do have to be cautious*…*I feel*, *like*, *it’s amplified more here*, *in Georgia…And it’s crazy because even when I travel to other places like LA*, *or Philadelphia*, *or*, *like*, *it’s*, *it’s*, *not*, *it’s not like Atlanta…give me all the PrEP… I got to make sure I’m doing what I got to do if I’m going to continue to live here…*

(Jay)

Still, others highlighted how the HIV response directed towards YBGBM in Atlanta by cultural outsiders perpetuated stigmatization and fatigue within the community. Participants believed that the intense efforts reduced Black gay men to their sexuality and HIV status, thereby further marginalizing them and distancing them from PrEP and other forms of HIV prevention. For example, one community organizer described how the widespread use of the term “MSM” perpetuated stigma and exclusion, stating:

*Public health authorities have conflated Black gay men—an identity*, *with a risk category*. *‘MSM’ is offensive because it takes Black gay men out of history*

(Provider C).

Another described the unintended yet deleterious consequences of HIV fatigue on mistrust and PrEP engagement. When asked about barriers to PrEP use among clients, he stated:

*Medical mistrust is a barrier*. *Young men have told me that they feel like they’re being targeted by health campaigns…they feel like everyone is trying to make money off of them*. *It stigmatizes them by focusing on HIV*, *instead of other issues that impact them*.

(*Provider* A)

#### Scarcity of medical and community resources to address unmet mental health needs

Themes about intersectional stigma often centered on the unmet mental health needs and scarcity of mental health resources available to support coping with trauma and other mental health conditions. When discussing community-level barriers to PrEP, one participant described how the burden of intersectional stigma and racialized inequities contributed to depression and a sense of apathy among some YBGBM in Atlanta when it came to PrEP and HIV prevention-related matters:

*We are already depressed people because of our color*, *of being black*, *period*. *Do you have to worry about you being gay and who’s going to accept you*?…*You have stigma over you being a gay*, *black man…that’s what makes a lot of the black people not want to deal with it…white people have more advances…They have more things than the black community will ever have*, *which makes it a lot easier and more accessible for them to do PrEP*.

(Jay)

Another described the crucial importance of mental health care to PrEP continuation:

*The mental health care is horrible…they need more mental health care for down here*. *But what was always pushed was the PrEP*. *So even though I didn’t have the pills that I have been on for years for mental health*, *they* [providers] *made sure I got a new prescription for these* [PrEP]*…It’s something missing and I really think what’s missing is the bridge between*, *‘Here’s your PrEP*, *go do what you want*,*’ which that’s what it is down here because in Chicago you got to talk to some people before they let you get PrEP…*

(Lamar)

Despite holding a common view of Atlanta as a cultural mecca for Black gay men, providers and clients expressed a need for recreational outlets for young Black men to share culturally affirming support to support mental health and care-seeking. For example, when asked to share his viewpoint on resources to support the health of YBGBM in Atlanta, a provider stated:

*I’d like to see more things focused on emotional wellness vs*. *sexual health*. *Did you know that there are no public spaces to support agency*, *identity development*, *and constructive activities for Black gay men?*

(Provider C)

The significance of recreational outlets was further stressed by those who were on PrEP. Such as the following statement, in which a participant described how his mental health affected PrEP use and sex:

*…if*, *you know*, *you had a fucked-up day*, *you had somebody cuss you out*, *you lost your job…you can go to the event that bathhouse*, *err*… *That’s your only outlet*. *And that’s how it is for a lot of Black*, *gay guys… So*, *you’ve got to think about all those aspects of the gay*, *gays that live here who go through very traumatic experiences and are going to stick with something that makes them feel good*.

(Jay)

Another stated:

*…Everybody goes to different points in their life*, *and I actually have been it at that point in my life where I was*, *like*, *willing to risk it all*, *‘I didn’t take my PrEP today*, *but I don’t care’… I mean*, *I had hook ups*, *but for me*, *it was like a mental thing*, *because I was so unhappy with my own regular life*. *And now at this point*, *I wasn’t getting the medication I needed*. *So then*, *what do I find*? *I found an outlet and the only medication I was taking was something that was allowing me to go hop in the bed and forget that I had issues*.

(Lamar)

### Interpersonal

#### The importance of provider support

On the interpersonal level, we found that nurses, medical providers, and PrEP coordinators shape and determine PrEP access through eligibility criteria, PrEP education, patient referral practices, and the provision of ongoing support. In the excerpt below, one participant describes the importance of screening questions during routine visits:

*Interviewer*: *What are some of the reasons why you acted on getting it [PrEP]*?

*Participant*: *It was I think a year*, *two years ago*. *I’d gotten really sick*, *but it wasn’t for like anything sexual or anything*. *I was at the hospital; they did some blood tests*, *and my doctor actually recommended it for me because of answering a few questions or whatever*. *I always knew about PrEP*. *I just never thought about getting it*, *and so I just decided*, *‘Okay…I might as well just do it.’*

(Brandon)

Overall, clients described having positive experiences with local providers. When describing his experience with providers, a participant stated:

*Like*, *I say that they’re really personable…As I could tell that they don’t really have any*… *judgment or like bias…it’s kind of*, *like*, *a judgment-free zone*. *That kind of helps to really cultivate a really safe space*.

(Malcolm)

Clients expressed gratitude for appointment reminders and advice from providers, as well as a quick response to challenges. For example, when asked, *how would you describe your relationship with your provider and your experience with them*? A participant replied: *It’s been nice…*. *Most of the time when I call them*, *if they aren’t able to pick it up*, *they will call back pretty soon*. *They have information*, *like*, *the tip of the bill* (James)

Another described appreciating being nagged about appointments. When asked about his PrEP care experiences with providers, he replied:

*I do feel cared about because you know*, *they’re reaching out to me…They keep up with you*… *and they want to make sure that you’re*, *you know*, *kept up with your labs and all that*. *And so*, *I feel like that influences me the most to continue taking it…I do like feeling*, *like*, *they actually have my back*. *Like*, *they are the ones annoying me*. *I’m not annoying them about it*. *Because I tend to sway and so*, *you know*, *being on track is important for me*.

(Brandon)

Provider described the measures that they took to support positively affirming client interactions. When describing her experience working with YBGM, one affectionately referred to her clients as ‘*my babies*.’ Other providers detailed how they exercise care with the terminology that they used when interacting with clients stating: *We try to de-medicalize our health promotion efforts*, *so we refrain from using stigmatizing terminology such as*, *‘risk’ or ‘eligibility criteria*.*’* (Provider B).

One important communication gap between providers and clients was highlighted in discussions surrounding PrEP adherence. All clients described missing doses when asked about their PrEP use patterns, and one provider discussed HIV seroconversion among YBGBM male clients as a prominent personal concern. When probed further about knowledge and provider discussions on how to restart PrEP after missing one or more doses, none of them knew what steps to take to restore the clinical effectiveness of the medication. The following excerpt signifies the confusion that YBGBM experienced when it came to missing doses:

*Interviewer*: *What do you do when you forget to take it*?

*Participant*: *I’ve actually thought about that*. *Like…if you miss a day*, *do you take it twice the next day*? *I just never had that conversation because… I don’t know—like I don’t even like taking pills anyway*. *So*, *I didn’t really…care to ask*, *“should I do this*, *or should I not do this*?*” Because I just put the bottle down and just*, *you know*, *kept pushing*.

(Brandon)

When asked a similar question, another participant brought up the possibility of having designated PrEP community liaisons or sponsors to check with for information needs:

*I think also a big thing is maybe*, *you know*, *people who aren’t getting maybe a designated provider or a designated mentor*, *designated health professional*, *that directly deals with that person’s*, *you know*, *PrEP*, *that they can trust from the jump to be like*, *you know*, *this is my PrEP person… just somebody that you can call…they can pick the work phone up and be like*, *“Hey*, *everything good*?*” Especially in those communities of color…people of color trust people that they got history with…*

(Lamar)

*The influence of partners and relationship factors on PrEP use patterns*

Participants described how changes in relationship status and sexual activity influenced PrEP use. Those in monogamous partnerships described discontinuing PrEP at some point in the relationship, saying something like the following participant: *I was on it for about three months and then*, *we like*, *officially made it kind of sort of closed the relationship…we both jumped off of it*. (Devon). Yet, partner trust and fidelity were primary concerns and determinants of starting or stopping PrEP. Bringing up PrEP or using it within a monogamous partnership was viewed as complicated and conflict-ridden because it raised suspicions about stigma, infidelity, or lack of trust. For example, a participant described the stigma and fidelity concerns associated with mentioning PrEP to a partner after they began a relationship:

…*if you would have been talking to somebody for a while…but then all of a sudden*, *now we’re sitting down*, *we’ve been in it for about a couple months…that conversation is a little awkward*. *It’s taboo*. *Like*, *are you asking me…’Do you feel like I’m a hoe?*

(Lamar)

In a different scenario, a participant described how his friend hid and eventually discontinued PrEP due to fears of compromising partner trust:

*One friend*, *he was on PrEP*, *but he ended up getting off because his boyfriend didn’t know he was on it*, *and I was like*, *‘why do you keep it a secret*, *you know*, *that you are on PrEP*?*’ I didn’t get the hiding part*. *It could be misconstrued as*, *‘I don’t trust him*,*’…So when he ran out of pills*, *he just didn’t take them anymore*.

(Corey)

Another who used PrEP in a past open relationship stated the following when asked how PrEP use might change in future relationships:

*…If I was in a monogamous relationship…I would definitely say it would change*, *as we expectedly wouldn’t be having sex with people…It probably shouldn’t change because things happen*, *people slip up*, *they might cheat*, *and you just hate to be in a compromising situation*. *But I’ll definitely say*, *in relationship*, *I’d probably be less likely to take it*.

(Malcolm)

### Individual

#### The role of developmental stage and traits

Emerging adulthood traits, such as residential mobility, life transitions (e.g., employment, work, and school), as well as, identity exploration and development [28] were individual-level factors that collided with structural barriers surrounding ease of access to PrEP. Participants mentioned moving often. For example, one participant described transferring care three times during his young adult years, stating: *I had PrEP in three different states* (Lamar). As busy young adults who were navigating relationships, jobs, and school, they also experienced frequent fluctuations in their social and sexual lives. For example, when asked about his PrEP use a participant replied:

…*I’ve kind of fallen off recently*. *Only because I’ve been just so busy and then I was out of town for like three weeks… So*, *I just knew I wasn’t going to be sexually [active] anyway*.

(Brandon)

Providers also brought up care retention and PrEP adherence challenges that they encountered with young clients. One spoke about frustrations with retaining people between the ages of 18-and 25 years in PrEP care for more than 6 months.

*We have to constantly call these patients for one to two months in order to get them to come back in for clinicals…When we reach some*, *they say that they don’t need PrEP anymore because they have changed their behaviors*, *but a few ended up seroconverting…*

(Provider A).

The same provider later acknowledged the misfit between clinical hours (9 am– 6 pm) and young client, saying, *young people want to be able to come in and get PrEP when they want it* (Provider A).

Another provider described often receiving emergency medication refill requests from younger clients. While recalling trying to get them medication with little notice she said, *the young ones usually call me when they are down to one pill right before closing and before the weekend*. (Provider E).

Another emerging adult trait that was presented in discussions about PrEP use was low efficacy in managing PrEP use and life challenges, in general. This was expressed in a previous quote in which “Brandon” noted his inconsistency with PrEP use along with other things in his life. Similarly, while discussing his reservations about starting PrEP, another participant said:

*Participant*: *I’m uncertain in a lot of things… I’m unsure in all areas of my life*.

*Interviewer*: *Where do you think that kind of comes from?*

*Participant*: *Just being young*.

(James)

#### Generational distance and culture

Adjacent to life stage characteristics was the generational distance between YBGBM and the acute HIV epidemic. Participants believed that the perceived threat of HIV among YBGBM men had declined over the years, in part, due to seeing people manage HIV as a chronic health condition. For example, when asked about PrEP use in past relationships, a participant said:

*I am a millennial and I’m young… I got so many different friends who have HIV* [and] *I was with somebody who was HIV*. *So*, *it was like*, *it’s becoming*, *um*, *like a new normal*. *Once it potentially hit me and I have syphilis and I didn’t know if I had HIV either…that’s when it was like*, *‘Oh*, *I got to take this seriously.’*

(Jay)

Participants also believed that cultural and historical health care relations additionally factored into PrEP care engagement among YBGBM along with life stage. Other participant accounts likewise described the direct and indirect impacts of medical racism on care-seeking and skepticism. In describing his experience with discussing PrEP with a provider, a participant said:

*I was very pessimistic about it…I was a non-believer …I mean, my grandma was born in 1936. She is from down South. There was no Health Care…We [communities of color] are on our last leg before we decide to go to the hospital…we would sit there and die in our living room on our last breath before we decide to go and take ourselves to the hospital…*

(Lamar)

The statement regarding medical care as a last resort aligned with the accounts of participants, who described getting on PrEP only after being pushed into a clinical setting by HIV exposure or a different health-related matter. These situations were crucial to shifting health risk perceptions, magnifying the importance of preventive behavioral measures, while also placing them in a position to be screened by a provider. Providers explained that STI treatment and emergency medicine were primary referral avenues. Other participants provided perspectives concerning the lack of motivation that they felt about engaging in preventive health care as healthy young men. For example, when asked to reflect on reasons why he did not move forward with pursuing PrEP after hearing about it through multiple avenues, a participant replied:

…*It’s like the old saying*. *‘Don’t fix something if it ain’t broke*.*’ And I feel*, *like*, *a lot of guys they wait until scare happens*. *They’d be like*, *“Okay*. *Sure*. *Let’s do it.”*

(Brandon)

#### Concerns with side effects

The participant above also described how his health emergency reduced his concerns about the side effects—which was also a common concern and barrier to PrEP initiation and continuation in the early stages of uptake among participants.

*I knew about the side effects…That was actually one of the biggest factors for me…I was like*, *“I don’t want to take this*. *Like no*.*” But I don’t know*. *I guess that just one moment of like being super sick just was like*, *you know what*? *Forget about that…*

(Brandon)

When describing his day-to-day activities, one provider described spending most of his time addressing fears and myths about medication side. When asked about his hesitancy with trying PrEP another participant said:

*I was kind of hesitant at first because a lot of people turned out*, *like*, *you have like stomachaches and headaches and things like that…I’m like*, *I don’t know…*

(Corey)

Other participants mentioned switching brands when after experiencing side effects. A participant with kidney health complications described how fearful he was while taking

*… the doctor found out about Truvada being too harsh on my kidneys three months later. So, I was like "Oh, my God! [Laughing] I’ve been taking this for this long and you’re just now telling me this?” … So, it was very difficult to, to stick with it. After that, when they put me on Descovy, that’s when it, it just got a little bit more difficult for me too want to trust the medication*.

(Jay)

#### The biomedical effectiveness of PrEP

Protection against HIV transmission strongly motivated PrEP use despite the challenges that participants experienced. Taking a daily medication was not viewed positively, but all participants recognized the benefits of being on PrEP. These benefits included its effectiveness in blocking HIV, thereby reducing fears and inhibitions about contracting HIV during sex, while also promoting peace of mind. When participants were asked about what they liked about PrEP, most responded like the following:

*I mean*, *it’s a pill*, *so really the safety of it all*. *I just can feel more at ease about it*. *It’s crazy levels in our community*. *It makes me feel safe…*

(Malcolm)

Another described it as a sexually empowering two-step verification method when used with protective barriers:

*Interviewer*: *What do you like the most about taking PrEP*?

*Participant*: *… feeling like*, *we have our two key authorization going into sex [Chuckles]*. *Like Facebook and Twitter*, *you make sure you wrap it up and take your medication*. *So*, *it’s a double barrier that makes me feel better when I am out here*

(Jay)

## Discussion

The primary goal of this study was to identify multilevel PrEP use determinants among YBGBM, with the main objective of addressing knowledge gaps that will drive the development of EHE HIV prevention equity strategies in a high-priority jurisdiction—the metropolitan Atlanta area. Through collaborative research-community research, we have generated recommendations that center the needs of YBGBM in the distribution of resources, and development of programs and policies that may support PrEP equity. Additionally, the ethnographic process, which fostered the iterative engagement community members, generated insights into the social lives of YBGBM to contribute a holistic view of how they navigate PrEP decision-making and use within the context of emerging adulthood, relationships, and identity development. A discussion of the specific recommendations within the context of our findings and the broader literature follows below:

### Recommendation #1: Flexible PrEP care and prescription access modalities are needed for linking and retaining YBGBM in PrEP care

The setting, location, and means by which PrEP could be accessed were structural factors that often intermingled with the intersectional stigma to influence PrEP use. Limited options for accessing PrEP are structural barriers that can be eliminated by promoting multiple pathways for getting and retaining YBGBM on PrEP. Our data suggest that extended clinical hours for accessing PrEP are needed. Additionally, mail delivery programs are one option that may work well for stably housed YBGBM in Atlanta, especially among those who are deterred by HIV stigma. This recommendation may be particularly relevant to providers who also offer HIV treatment. It is important to note that mail delivery programs may not be beneficial to people without a permanent home address or private mailbox. Continued investigation into alternative PrEP care pathways, aside from mail delivery and clinical programs may support the development of additional options for facilitating PrEP access among YBGBM.

### Recommendation #2: Increased investment in medical and community-based mental health resources to stem and account for the negative effects of intersectional stigma (social identity, HIV, and spatial) on YBGBM in Atlanta

Using an intersectionality theoretical lens enabled us to identify the axes of intersectional stigma (Berger [29]) that uniquely shape PrEP-seeking and adherence among YBGBM in Atlanta. Our findings agree with literature identifying intersectional stigma as a powerful and pervasive determinant of PrEP use among BGBM [30]. While the motivational aspects of intersectional stigma on PrEP adherence surprisingly contradict research demonstrating its negative effects on HIV prevention method seeking, it is possible that YBGBM in Atlanta who are not living with HIV (such as those in this sample) may be experience HIV stigma by association due to their close proximity to others who are living with HIV or are an unknown HIV serostatus [31]. For example, when examining HIV stigma by association among non-HIV-positive Australian GBM in 2020, Broady, Brener [31], found that nearly 72% of GBM had felt stigmatized by people who assumed that they were at risk for HIV, with 17% often or always experiencing stigma by association. Additional results indicated that those with more experiences of stigmatization by association had higher HIV-testing rates, more experiences with stigma, and felt more strongly connected to the GBM community. Thus, stigma by association is a concept that may complicate PrEP seeking and use among non-HIV-positive YBGBM in Atlanta, who are navigating HIV stigma due to their social membership, though they are not living with HIV. Additional research, perhaps with a different study design and larger sample size is warranted to gain an in-depth nuanced understanding of stigmatization types and mechanisms that affect PrEP-seeking and adherence among YBGBM in Atlanta. Nonetheless, our data clearly indicate a need and strong desire for resources that stem the sources and negative impacts of intersectional stigma on YBGM. Link and Phelan [32] identify power as the source of stigma, stating that “stigma exists when elements of labeling, stereotyping, separation, status loss, and discrimination occur together in a power situation that allows them. Thus, while our observations are at the individual-level, they ultimately point to power imbalances that breed intersectional stigmatization through social inequality. YBGBM participants’ acute awareness of intersectional stigma as manifested through a lack of accessible mental health care resources, skepticism and distrust of medicine due to racism, and the magnified emphasis on HIV (vs. other social and health needs) among YBGBM cannot be ignored in local PrEP equity efforts. Policy and community-level stigmatization reduction interventions that stem the sources of racism, homophobia, as spatial stigma, particularly within social, public health, and healthcare delivery settings are needed to facilitate equitable access to PrEP among YBGBM. As such efforts are undertaken, increased access to competent and trauma-informed mental health care is essential important to mitigate the harms of racial trauma and other minority stressors on YBGM. The integration of routine psychiatric screening before and during PrEP care may be crucial to supporting care retention and medication adherence along crucial aspects of health and wellbeing [33]. Finally, the unparalleled pride, solidarity, resourcefulness, and ingenuity of the YBGBM community in Atlanta, as found in this ethnographic study strongly suggest a potential for reaping PrEP and social equity through an increased economic investment in local community-based organizations. In Atlanta, community-based organizations and networks that are developed and led by and for GBM serve as sites of culturally affirming and relevant support through recreation activities. Investment into these organizations can significantly improve mental health outcomes and PrEP use.

### Recommendation #3: Community education resources are needed to increase understanding of (1) financial assistance programs and (2) how to recover HIV protection after missed PrEP doses

The assessment revealed a need for increased awareness about financial assistance programs for PrEP care and prescriptions. Health professionals should address these barriers when seeking to engage clients and their networks in PrEP care. Other promotional programs and resources directed toward informing YBGBM about PrEP in Atlanta should likewise provide detailed information on how YBGM can receive PrEP for free or at reduced costs.

Confusion on how to use PrEP after missing one or more doses was a prevalent concern that signified a need for improved PrEP patient-provider communication. This finding is especially concerning given PrEP users’ descriptions of intermittent PrEP use, due to life disruptions or changes in their sexual activity and relationship status. Discomfort with discussing sex behaviors, insufficient knowledge about how PrEP works and time constraints are a few communication barriers that have been highlighted in other research [34]. There were no data to suggest that these conversations did not occur due to the discomfort with discussing sex and PrEP use patterns in this assessment. With few exceptions participants described positive patient-provider interactions. Research exploring barriers to PrEP use among YBGM in other regions contrasts with these findings. For example, when exploring the healthcare experiences of Black GBM in Wisconsin, researchers found that passive-aggressive racism, patient-provider racial discordance, and homonegativity within medical establishments were prominent barriers to PrEP use [35]. In comparison, our findings about provider-patient interactions this assessment are encouraging, especially given the clear concern that providers expressed for their clients, and the care that they exercised with rejecting stigmatizing terms (like “risk”) that have previously been found to be negative aspects of GBM PrEP patient-provider interactions in Atlanta [36].

### Recommendation #4: Adaptive patient care models are needed to implement patient-centered PrEP care for YBGBM men in Atlanta

We identified multiple cultural, relationship, and life stage developmental traits which influenced PrEP use and care engagement. The participants in this study were in the stage of emerging adulthood. Residential instability/mobility, exploration, and a “subjective sense of ambiguity” are core defining developmental characteristics which are associated with this stage [28]. Our data confirm that these characteristics often clash with traditional and rigid PrEP delivery models. While some providers were frustrated and confused about PrEP attrition among YBGBM clients, the ground-level view on the lives of YBGBM PrEP clients gives a powerful counternarrative that shifts the primary determinant from clients who are more likely to experience life disruptions due to transition to a system of care that does not adequately accommodate the needs of this group. There is some recent data to suggest that mobile or network-oriented PrEP interventions may be an innovative way to support PrEP uptake and adherence among YBGBM [37, 38]. Most gay male participants in San Francisco and Chicago who participated in a mobile health PrEP adherence support intervention pilot believed that the messaging intervention was feasible and acceptable. The study authors also found the highest acceptability for PrEP check-ins via text messages among younger and non-white participants [37]. Importantly, the intervention exhibited preliminary effectiveness in reducing missed doses by 50% among participants (Fuchs, 2018). Ongoing PrEP support may also present alternative means for PrEP clients to communicate about their concerns with side effects or any other insecurities or challenges that come up when taking PrEP. The data from this study additionally emphasize the importance of future research and interventions to address the role of cultural and historical healthcare injustices and beliefs on PrEP care-seeking among YBGBM. While exploring PrEP awareness and uptake with focus groups with BGBM in the south, researchers likewise found medical skepticism and mistrust to be barriers to PrEP seeking among participants [39]. The camaraderie between the researchers, providers, and community leaders who supported the development and implementation of this assessment provides strong evidence for a potential pathway to address medical skepticism and mistrust through alliances between health professionals and community leaders.

### Limitations

We acknowledge the limitations of the study due to the design, sampling methods, and project constraints. Time and resource limits are definitive features of REAs. Due to such constraints, we were unable to audio-record provider interviews. Despite detailed notes and quotations serving as records of the provider interviews, important information may have been inadvertently misconstrued or excluded. Selection bias is another limiting factor that may have constrained the diversity of perspectives concerning PrEP determinants. An attempt was made to use snowball sampling—a peer-referral method commonly used in qualitative research, to recruit additional participants; however, all referrals came from providers. Sampling PrEP users through providers may have led us to capture more clients and providers with positive experiences while excluding those who may have avoided or dropped out of PrEP care due to negative experiences. There may be additional selection bias due to the use of videoconference interviewing methods. While this concern may not have affected provider inclusion, it may have limited the inclusion of PrEP users who are without access to those resources. Finally, interviewer bias due to the discordant race and sex of the interviewers and participants may have also influenced participants’ responses to interview questions—particularly those on sensitive topics, in addition to the interpretation of the data on behalf of the interviewers. To mitigate against the impact of discomfort resulting from this bias, participants were told about the sex and race of the interviewer during eligibility screening and allowed to select the interviewer whom they expected to be most comfortable with.

## Conclusion

PrEP uptake among YBGBM must substantially increase to successfully bring about an end to the HIV epidemic in the United States. In short, the traditional models of care which work well for non-black, middle-aged, and heterosexual people are ill-fitted to address the needs of YBGM—who may use PrEP more effectively if given the flexibility and expanded clinical hours and education about how to use PrEP flexibly. There is also a need for resources that holistically focus on mental health, trauma, and racism as critical components of PrEP support. Making PrEP programs more responsive to the lived experiences and expressed health care needs of YBGBM can contribute to increasing PrEP uptake and adherence within this community.
